# Candiduria in ICUs: incidence, course and outcome

**DOI:** 10.1186/cc11706

**Published:** 2012-11-14

**Authors:** D Juneja, P Nasa, O Singh, Y Javeri, S Garg

**Affiliations:** 1 Max Super Speciality Hospital Saket, New Delhi, India

## Background

There is a high incidence of candiduria especially in ICU patients. Candiduria may be a sign of a disseminated candida infection and a marker for increased ICU mortality. However, there is always a dilemma regarding which patient to treat, as in a majority of patients it may only be a colonizer. Moreover, the choice of antifungal drug is also controversial due to low urinary concentration of many antifungal drugs. Hence, it becomes imperative to have knowledge of locally prevalent species to guide treatment protocols. The aim of this study was to assess the incidence of candiduria among patients admitted to a medical ICU of an Indian hospital, to perform microbiological characterization and to study their ICU course and outcome.

## Methods

Data from 93 consecutive ICU patients with candiduria, admitted during an 18-month period, were obtained retrospectively. Data regarding patient demographics, ICU course and outcome were entered in a *pro forma*.

## Results

Out of 3,142 ICU admissions, the incidence of candidemia was 29.6/1,000 admissions. A high proportion of patients (80.6%) had an indwelling urinary catheter with the mean duration of catheter days being 5.89 ± 3 days. Other associated risk factors such as diabetes mellitus and antibiotic usage were seen in 74.2% and 86%, respectively. Concomitant candidemia was seen in 19.4% of cases. Nonalbicans Candida spp. (66.7%) emerged as the predominant pathogen causing candiduria. ICU mortality was 29% (Table 1).

**Table 1:** Patient demographics, ICU course and outcome

Parameter of interest	Frequency
Age (years) (± SD)	59.81 (± 19.5)
Sex, males (%)	54 (58.1%)
Days in ICU before positive culture (± SD)	10.11 (± 9.8)
Presence of urinary catheter (%)	75 (80.6%)
Days since urinary catheter was present (± SD)	5.89 (±-3)
Admission APACHE II score	17.13 (± 7.8)
Predicted death rate (%)	30.36 (± 21.1)
Previous antimicrobials (%)	80 (86%)
Recent hospitalization (%)	7 (7.5%)
Neutropenia (%)	3 (3.2%)
Nonalbicans infection (%)	62 (66.7%)
Blood culture positivity (%)	18 (19.4%)
Need for vasopressor support	52 (55.9%)
Need for renal replacement therapy	25 (26.9%)
Need for mechanical ventilation	60 (64.5%)
ICU length of stay (days)	21.34 (± 23.3)
Hospital length of stay (days)	27.38 (± 25.2)
ICU mortality (%)	27 (29%)

## Conclusion

There is a high incidence of candiduria in ICU patients, especially among those with indwelling catheters and those on antibiotic therapy. Moreover, in our cohort, an increased proportion of patients with candiduria had noncandida infection, emphasizing the need to have localized regional surveillance studies to identify the locally prevalent candida species and devise antifungal therapy protocols.

